# Establish a machine learning based model for optimal casting conditions management of small and medium sized die casting manufacturers

**DOI:** 10.1038/s41598-023-44449-0

**Published:** 2023-10-11

**Authors:** Sangwoo Park, Sekyoung Youm

**Affiliations:** Department of Industrial and Systems Engineering, Dongguk University, 30 Pildong-ro 1-gil, Jung-gu, Seoul, 04620 South Korea

**Keywords:** Information technology, Scientific data

## Abstract

Die casting is a suitable process for producing complex and high precision parts, but it faces challenges in terms of quality degradation due to inevitable defects. The casting parameters play a significant role in quality, and in many cases, producers rely on their experience to manage these parameters. In order to address this, domestic small and medium sized die casting companies have established smart factories (MES) and collected data. This study aims to utilize this data to construct a machine learning based optimal casting parameter model to enhance quality. During the model development process, distinct important features were identified for each company. This indicates the necessity of deriving tailored models for each site, aligning with the make to order (MTO) environment, rather than a generalized model.

## Introduction

Manufacturing based industries are inefficient structures with lower total component productivity compared to the high proportion of the national economy, and process technology underlying manufacturing is important for next generation manufacturing and competitive responses^1^. Process technology depends on the quality and performance of the final product, including casting, molds, welding, heat treatment, and surface treatment that give special functions to plastic processing and materials that manufacturing of the product, and surface treatment. This is called the root industry. This industry is indispensable for implementing technological capabilities needed to produce future oriented products such as eco friendly cars, semiconductors, OLEDs, robots, bio, and aviation and promoting preoccupation in markets. However, the international competitiveness of domestic manufacturing has been on the downward trend recently, and the proportion of process innovation is almost one-fifth of that of major European countries, making it even more necessary to innovate through the introduction of smart factories^1^.

Die casting is a process suitable for producing parts requiring complex product geometry or high dimensional precision by injecting molten metal into the mold cavity at high speed/high pressure, or has a problem of poor quality due to defects that inevitably occur during filling and solidification. In die casting, casting conditions are an important factor in determining the quality of castings, but in Korea, systematic research is insufficient, largely dependent on experience^2^. To solve this problem, many small and medium sized die casting companies in Korea have introduced smart factories (MES) to collect data from facilities and have a monitoring system. However, it is not being able to utilize the data collected due to problems at various manufacturing sites, only monitoring under what production conditions are produced.

A previous study related to quality data analysis and system application in the die casting industry has been conducted by Lee et al.^3^. In this study, this study developed a random forest technology based data analysis algorithm that predicts product defects by utilizing casting mold temperature data for foundries that produce automotive engine components, and applied the developed algorithm to the site. In addition, this study developed dashboards that can visualize poor prediction results and utilize those results to support plant operations. Even in manufacturing industries other than die casting, research has been conducted to predict the quality of products or diagnose the condition of facilities by utilizing data analysis techniques. Using the multiclass support vector machine technique, Widodo and Yang^4^ developed a diagnostic system based on the characteristic values of the facility’s fault data and claimed that the model could be applied to each component of the facility, including bearings, induction motors, compressors and turbines. Chen et al.^5^ developed an analytical model to predict the state of the facility using adaptive neuro fuzzy and high order particle filtering techniques. We demonstrate the superiority of the data analysis model developed by comparing the results of applying traditional machine learning techniques such as decision trees, support vector machines, and random forest techniques with the predictive accuracy of the results of applying the developed data model. Kim et al.^6^ developed a model that uses deep learning technology to determine defects in resistance point welding. As such, research on establishing and learning a big data analysis model based on machine learning deep learning is actively being conducted using manufacturing data. However, most of the research is centered on facility related data analysis studies, such as preservation of facilities and prediction of life expectancy.

Despite the presence of many production conditions management points, management is based on experience, and the criteria for setting labels that can be output to data analysis also depend on experience. To solve these problems, various studies from data collection system to analysis method are required. Based on this IoT based MES data, this study aims to establish models and solve quality classification problems by utilizing machine learning and deep learning technologies that have recently become a hot topic. In addition, to establish a general casting parameter classification model for die casting companies, a model was established that optimally classifies casting parameters by product using casting parameters data from two small and medium die casting companies.

### Data analysis based machine learning for manufacturing

Due to the presence of limitations in various manufacturing sites, research centered on process quality prediction has not yet been actively conducted. Predicting process quality has many specifications to consider and definition of causality is important. In response, the Korea Institute of Production and Technology^7^ understands the importance of process quality prediction and cause analysis and presents six items as follows: (1) Quality is often present at a later rank in process management because it is difficult to measure/evaluate compared to core competitiveness or production volume and visible indicators. (2) Quality measurement is often carried out after completion of the process, making it difficult to evaluate the quality level of the product during the process (3) Sampling measurement is carried out due to cost problems, and it is not highly related to the final quality of the product. (4) The introduction of a quality prediction system during the process shall enable preemptive quality control of the manufacturing process and early detection of defects. (5) Real-time monitoring of changes in process and quality situations through real-time data based quality prediction and feedback shall be possible. (6) A high-performance data mining model is required to learn the characteristics of process quality data such as large capacity and sample inspection. Therefore, in this study, classification models (deep learning and machine learning) were derived based on critical casting condition data in the die casting process to derive real-time data based quality predictions and feedback. This aims to reduce costs and promote high productivity by strengthening the ability of small and medium die casting companies to predict die casting process quality.

## Method

### Materials

Among various processes, research was conducted by limiting the quality of die casting processes within the casting process. The casting process consists largely of raw material injection, melting, die casting processes (mold setting/mold preheating, molten bath injection, low speed, high speed, pressurization, product extraction), post processing and finished product inspection processes. During the die casting process, simple repetitive work is causing problems of productivity improvement and frequent non operation and poor quality due to manual work by workers. The study focused on the problem of defectiveness.

Data from two small and medium die casting companies producing automotive components were utilized in this study. The first A Company produces electronic control unit (ECU) parts, which are automotive electronic control devices, using die casting techniques. After manufacturing and injecting molds and manufacturing products, workers’ visual inspection of defects on the exterior is carried out and the products produced daily are measured three times a day with a three dimensional measuring instrument to manage the quality of components. In other words, quality control is carried out through inspection after the entire process, but there was no quality determination standard in the die casting process, which is the main production process. To address this, we introduce MES systems and conduct real-time monitoring of casting parameters. However, The MES systems can only grasp the current status of casting parameters in the production site. There are limitations to identifying the optimal casting parameter conditions and improving productivity. Second, B Company is a company that produces parts for home appliances and automobiles through casting, processing, thought and assembly processes using materials such as aluminum, zinc, and nickel. Automotive parts manufacture ECU, bearing, radar housing and intercooler tanks. Home appliances manufacture gas boilers, gas ranges, induction, gas control valves, and rice cooker parts. Other products are manufacturing parts such as door locks and unmanned cameras.

**Figure 1:**
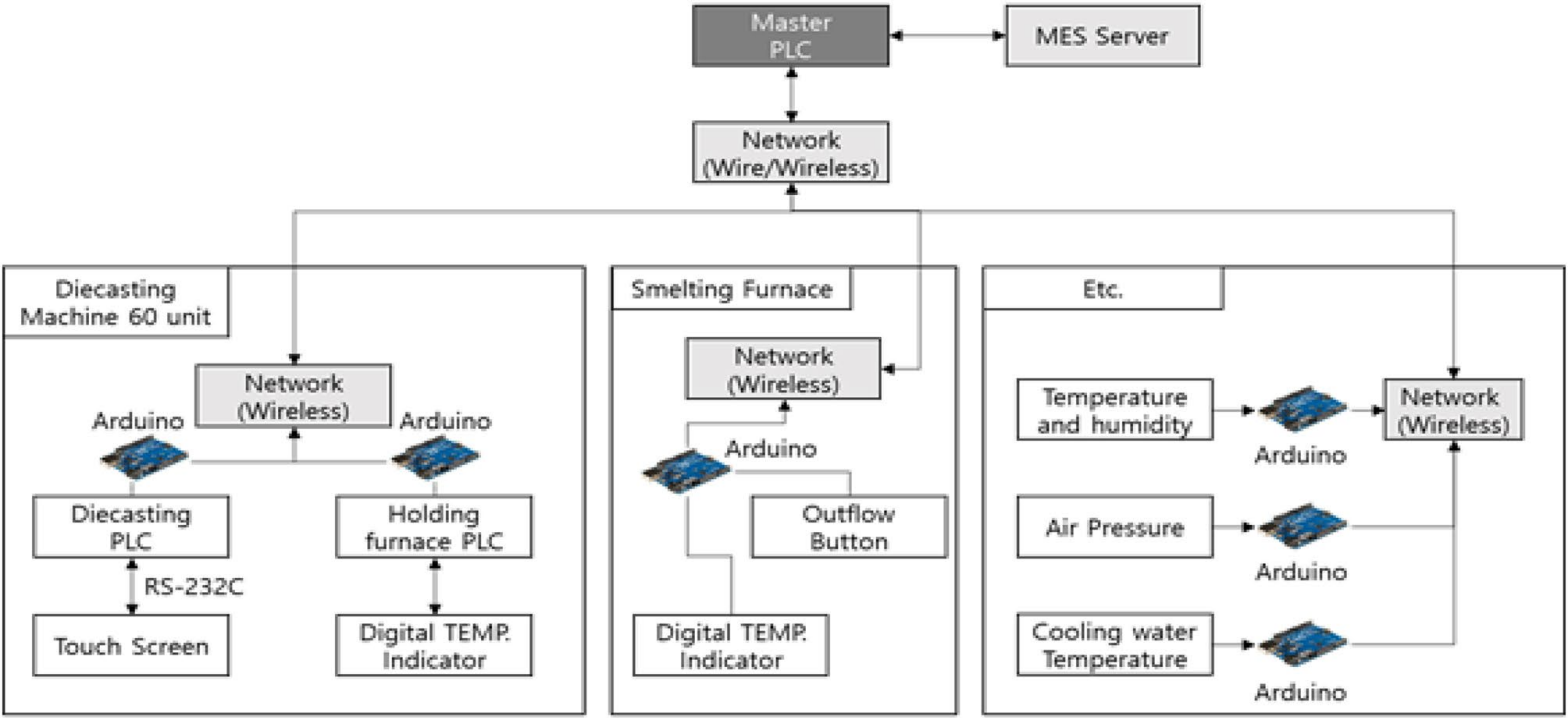
Data collection structure.

The facility data acquisition structure of two small and medium die casting companies is shown in Fig. 1. Information obtained from die casting equipment is largely divided into casting temperature/humidity, condition of the equipment, casting conditions (parameter), furnace/thermal furnace, etc. The temperature at the production site is a crucial factor that affects the raw material (metal) in the die casting process. It is utilized as a significant factor in quality since production conditions are adjusted according to seasons. Additionally, the temperatures of the holding furnace and melting furnace are considered important factors, as they ensure proper melting of the casting material and its maintenance in the appropriate state. The analog type facilities, furnace/thermal furnace data, and other data are converted into digital forms through arduino. The converted data is passed to the master PLC over the built network (wireless/wireless) and this information is sent to the MES Server. In this study, among the data collected by IoT, the analysis was conducted focusing on the data from the production facilities and the furnace data. Depending on the two small and medium die casting management elements, casting conditions are finely different, and models are established around casting conditions commonly managed by the two companies. Casting conditions are 1 speed (m/s), high speed (m/s), high speed (m/s), high speed (kgf), cylinder pressure (kgf), casting pressure (kgf), biscuit thickness (mm), low speed (s), high speed rise time (s), increasing pressure rise time (s), cycle time (s), high speed stroke (mm), high speed (mm), high speed.

**Figure 2:**
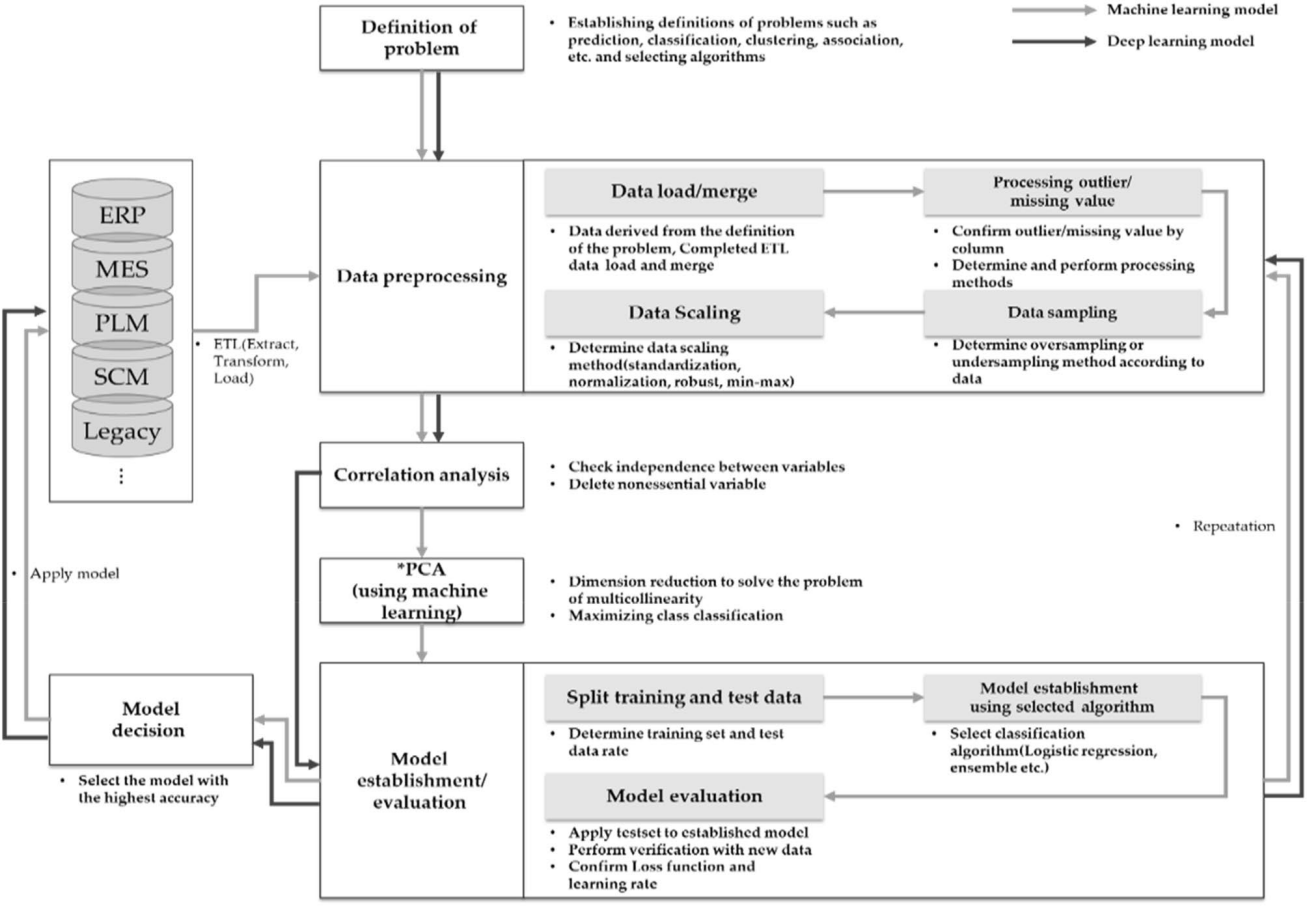
Model establishment process overview.

The process of establishing a machine learning model was carried out after problem definition, data pretreatment, correlation analysis, PCA (Principal Component Analysis), model establishment, and model evaluation. The process of establishing the model is shown in Fig. 2. Through the process of Fig. 2, a machine learning model utilizing data from two small and medium sized die casting companies was established and the model was verified by applying sample data from the product line.

In this study, the problem definition was defined as establishing a model that predicts quality based on product specific casting conditions in the die casting process to derive optimal casting parameter conditions as mentioned earlier. Since all three companies rarely can continuously utilize product molds in the MTO (Make to Order) method, they have established/derived models by separating product categories. In addition, die casting was limited to the data of TOYO V5 facilities due to the presence of casting parameter error by facility. The model was established and determined after preprocessing, correlation analysis, and PCA processes of the data. PCA was applied to solve the multicollinearity of each casting condition, and this method was used only to utilize the machine learning model. ANN models are established using low data rather than PCA.

### Data preprocessing

Performance is also essential to establish/perform an optimal model. In software engineering, various studies have been conducted on data pretreatment techniques to improve the performance of machine learning models. Typical data preprocessing techniques are divided into (1) Handling values, (2) Normalization, (3) Variable selection, (4) Variable construction^8–10^. In this study, data preprocessing process was defined as starting from data load to normalization, and variable selection and construction were performed at the PCA stage. By integrating data from each company, separating products, identifying the distribution of data by product (histogram, boxplot, etc.), and using InterQuartile Range (IQR) to process anomalies and missing values repeatedly.

The 2 year IoT data of the two die casting companies is 5,534,459 data, with the family of sensors, heat sink, powertrain, engine and fuel tank. bracket, home appliances, mufflers, battery equalizers, electric chargers, sealing plates, others, side mirrors, heaters, valves, connectors, antennas, and car doors. From here, only a family of over 10,000 data (sensors, heat sink, powertrain, engine, fuel Tank, bracket, muffler, battery equalizer, electric charger, and a total of nine) was extracted and preprocessed.

### PCA (principal component analysis)

PCA (Principal Component Analysis) is an approach that uses linear combinations to maximize the variance of the circle variables to obtain principal components that best describe various variables, and to see if changes in the circle are fully accounted for by some of them. The purpose of PCA is to (1) reduce dimensions, (2) explore axes with large variations, and (3) interpret data through principal components^11^.

**Figure 3:**
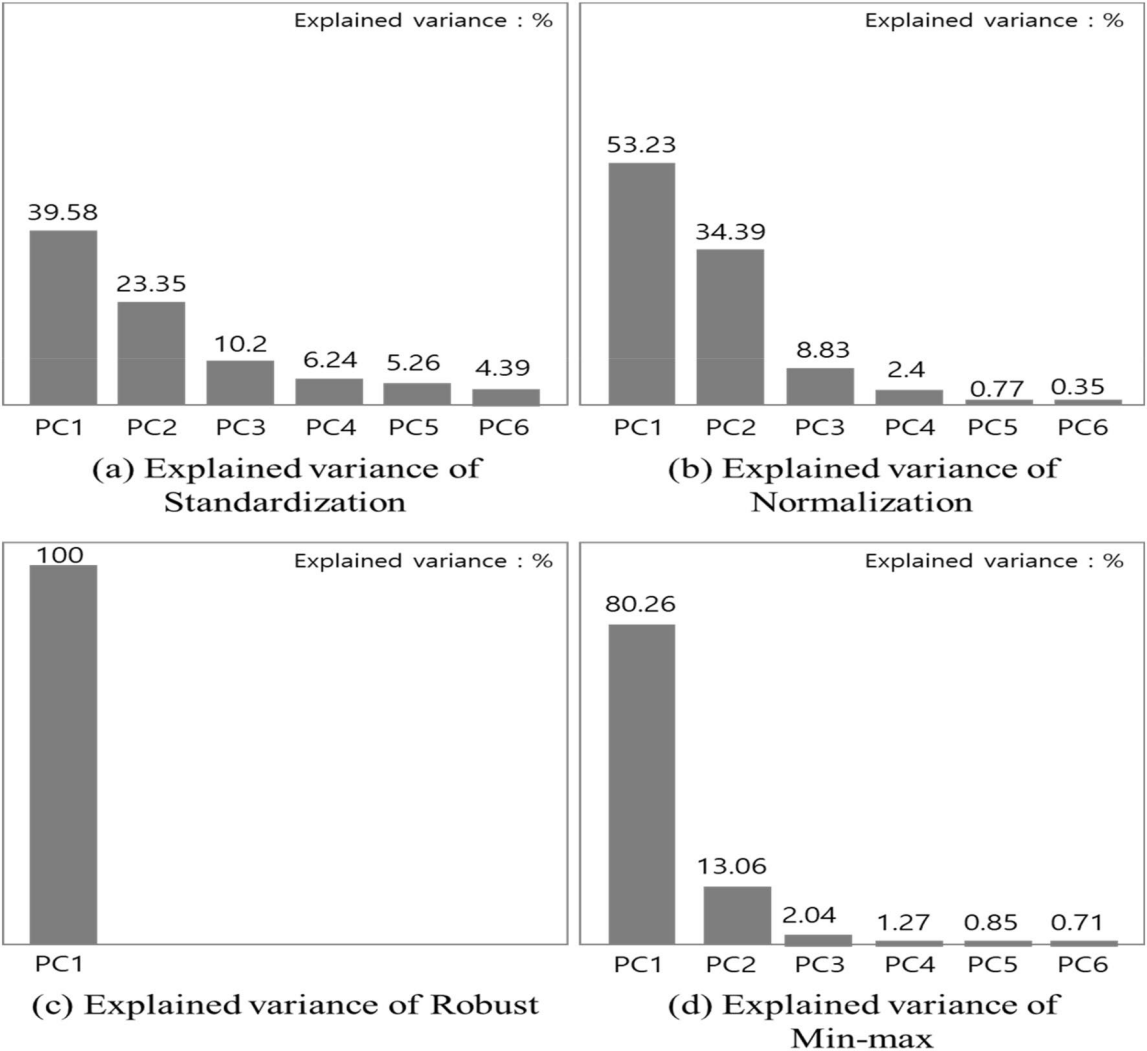
Explained variance by scaling method.

In this work, we applied linear PCA to reduce many dimensions of die casting and deal with the multicollinearity problem. Figure 3 is the result of performing a linear PCA using previously normalized data.

As shown in Fig. 3a, the PCA was performed with six components of standardization scaling data, and the explanatory power was PC1 (Principal Component1): 39.58%, PC2: 23.35%, PC3: 10.2%, PC4: 6.24%, PC5: 5.26% and PC6: 4.39%. As shown in Fig. 3b, the results of performing PCA with six components were PC1: 53.23%, PC2: 34.39%, PC3: 8.83% PC4: 2.4%, PC5: 0.77%, and PC6: 0.35%. PCA with six components of Robust scaling data, as shown in Fig. 3c, showed PC1: 100% explanatory power. One variable was shown to account for all variables. As shown in Fig. 3d, the results of performing PCA with six components were PC1: 80.26%, PC2: 13.06%, PC3: 2.04%, PC4: 1.27%, PC5: 0.85%, and PC6: 0.71%.

**Figure 4:**
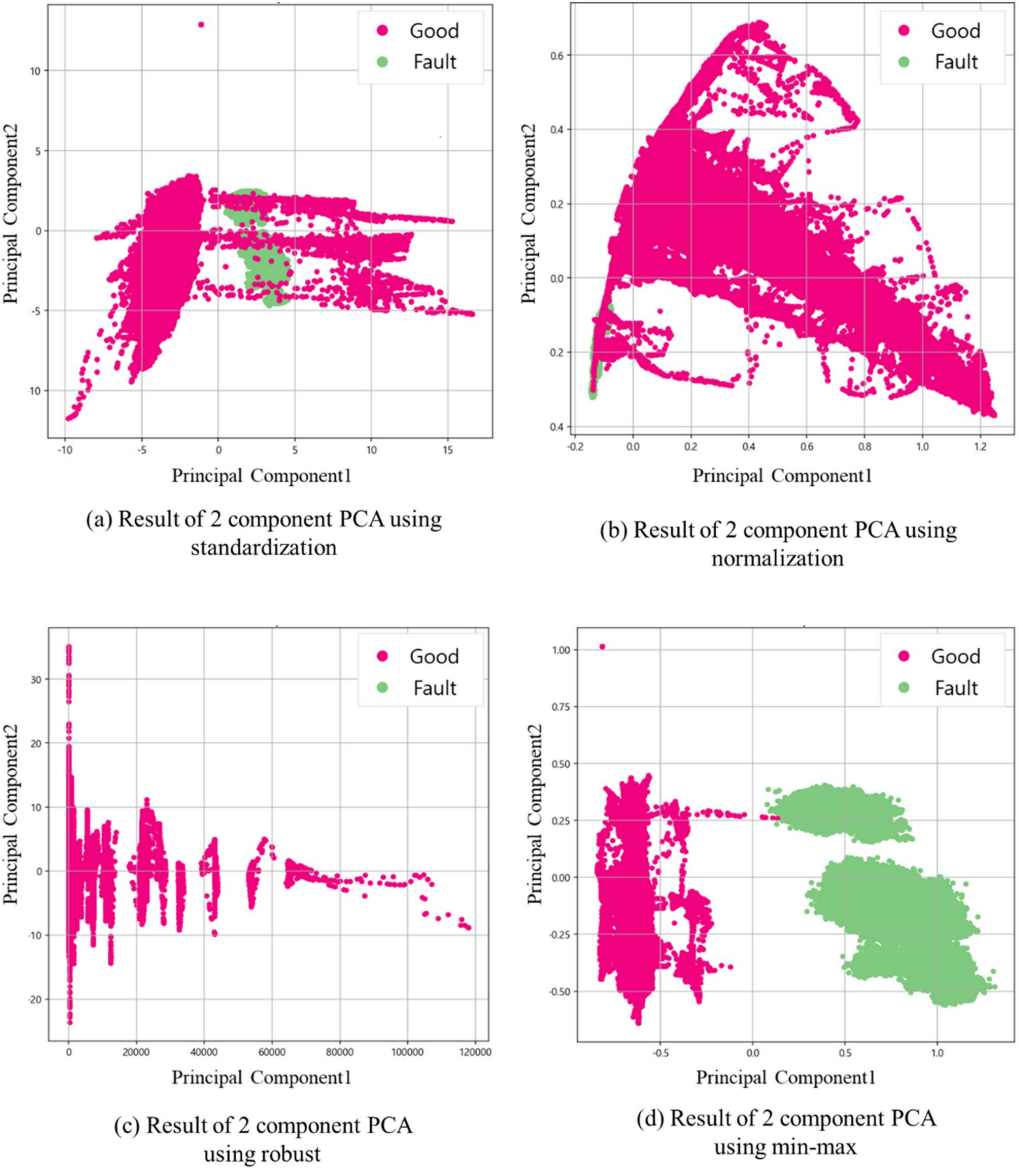
Result of 2 dimension PCA.

Standardization uses all six principal components, Robust uses one principal component and min–max uses two principal components, because the cumulative variance explanatory power usually selects a principal component that accounts for about 90%. Figure 4 is the result of performing PCA on two component criteria. When visualization was performed through two dimensional (using two variables), it was shown as min–max scaling technique that defect and good quality were completely separated.

### Model establishment/evaluation

A model was established using the overall data of two die casting companies, and logistic regression, ensemble, and ANN were applied as classification algorithms for model establishment. The current data acquisition state was assessed, and since labels exist, the problem was defined as a classification problem for predicting the quality status of the die casting process using supervised learning. Based on previous literature, algorithms such as logistic regression, random forest, and ensemble (including xgboost and gradient boost) were selected for solving the supervised learning classification problem. Depp learning algorithms (ANN), which are widely used in various fields, were also included comparative analysis^12,13^. A Validation was performed on the entire data model by using the data of the fuel tank product category randomly extracted from the established entire model as a test set. The model performed logistic regression, ensembles, and ANNs in order. Model data were divided into 70% training data and 30% Validation data. Machine learning techniques (logistic regression and ensembles) validated the model using PCA values as input variables. As mentioned, the PCA results were different according to data scaling values, so PCA was performed for all four data scaling types, and a model was established for each PCA result to derive the results. We compared the classification machine learning model using the deep learning accuracy to derive the most suitable model for Fuel Tank products categories.)

Logistic regression is a method used to classify two or more categorical groups or to predict the probability of occurrence of an event, with the aim of identifying the relationship between independent and dependent variables, such as linear regression, to select significant variables and build predictive models that can explain dependencies well^14^.

## Results

Results were obtained by applying the sampled fuel tank data to the Logistic regression, Ensemble model, and ANN model based on the overall data established earlier as a test set. The sample data were also applied as a test set by performing oversampling on the defect due to the significant difference in the proportion of good and bad products, similar to the overall data. It was derived that the accuracy of machine learning models was higher than that of ANN models, and that the accuracy differences were significantly higher according to the data scanning method when utilizing machine learning models.

### Logistic regression result

The values for logistic regression results collected from the entire data are given in Table 1. If you look at r square, Standardization, Normalization, and min–max data scaling represent the model’s explanatory power close to 1. Robust is 0.1022 and shows very low explanatory power. Standardization showed that PC3 and PC4 were not statistically significant (P > 0.05), and normalization showed that PC3 was not statistically significant. The results are given in Table 1.

**Table 1:** Result of logistic regression model using overall data.

Scaling method	R-square	P >|z|					
		PC1	PC2	PC3	PC4	PC5	PC6
Standardization	0.9799	0	0	0.973	0.902	0	0
Normalization	0.9895	0	0	0.121	–	–	–
Robust	0.1022	0	–	–	–	–	–
Min–max	1	0	0.413	–	–	–	–

The accuracy of the model was derived by inputting a test set using fuel tank data as a sample to the model established with the entire data. A total of 50,135 fuel tank data showed a large data imbalance between good and bad products, which resulted in oversampling of bad data with SMOTE techniques. The number of data that results from oversampling is 44,924 (a total of 89,848) good and bad products, respectively, and the results derived from this are shown in Table 2. Robust scaling data showed a very low accuracy of about 0.57. In addition, the Normalization scaling also resulted in a low figure with approximately 0.65 accuracy. On the other hand, Standardization scaling and min–max scaling showed very high accuracy of about 1.

**Table 2:** Logistic regression validation of overall ensemble model using fuel tank data.

Scaling method	Classification	Precision	Recall	F1-score	Support
Standardization (PCA4)	Good	1	1	1	44,924
	Fault	1	1	1	44,924
	Accuracy	–	–	1	89,848
Normalization (PCA 4)	Good	0.8	0.41	0.54	44,924
	Fault	0.6	0.89	0.72	44,924
	Accuracy	–	–	0.65	89,848
Robust (PCA 1)	Good	0.54	1	0.70	44,924
	Fault	1	0.14	0.25	44,924
	Accuracy	–	–	0.57	89,848
Min–max	Good	1	1	1	44,924
	Fault	1	1	1	44,924
	Accuracy	–	–	1	89,848

### Ensemble model result

The algorithms used in ensemble bagging models are logistic regression, random forest, k-nearest neighbors, adaboost, gradient boosting, and the accuracy of each model is as shown in Table 3. When min–max scaling was utilized, most models had near 100% results. All model parameter estimator has setting estimate = 50, except k-nearest neighbors (parameter k = 5). Also, overall model setting estimate = 50, k-nearest neighbors k = 5.

**Table 3:** Ensemble validation of overall ensemble model using fuel tank data.

Ensemble model	Parameter	Scaling method			
		Standardization (PCA4)	Normalization (PCA 4)	Robust (PCA 1)	Min–max (PCA 2)
Logistic regression	–	0.99991	0.65445	0.57090	1
Random forest	Estimate = 100	0.55102	0.49893	0.49996	1
K-nearest neighbors	K = 5	0.80741	0.68534	0.49993	1
AdaBoost	Estimate = 100	0.82411	0.56419	0.49741	1
Gradient boosting	Estimate = 50	0.70925	0.44071	0.49804	1
Voting	–	0.84246	0.48208	0.49804	1

### ANN result

The activation function (h()) utilized the relu function, and the σ() constructed the model using the sigmoid function. In addition, learning was performed by setting it to batch sizes 1000, epoch 18. The accuracy of ANN models established using 1,014,302 data was shown to be about 100% (0.999419). Statistical details of the model are given in Table 4.

**Table 4:** Accuracy of ANN model.

ANN	Precision	Recall	F1-score	Support
Good	1	1	1	1,014,302
Fault	1	1	1	1,014,302
Accuracy	–	–	1	1,014,302

**Figure 5:**
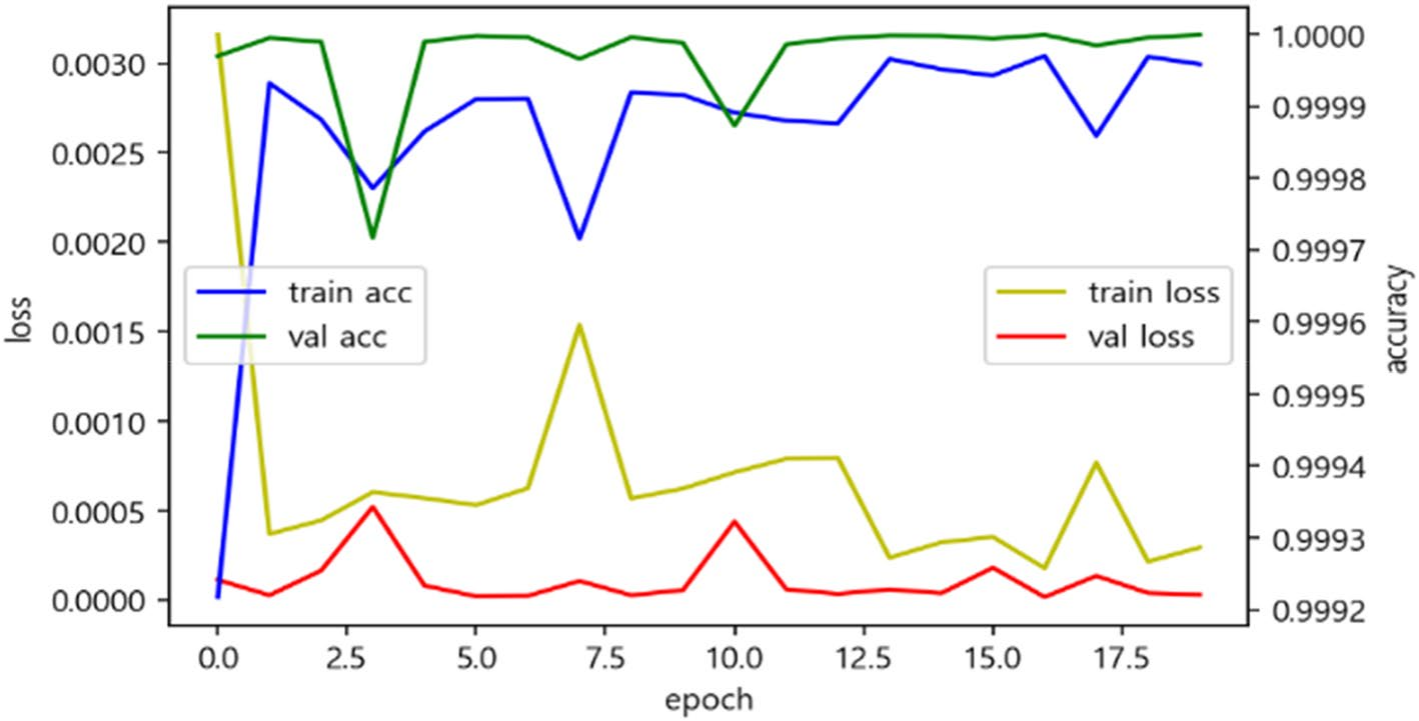
Loss, accuracy value of ANN by epoch.

Figure 5 is the result of learning progress, and most of the learning can be done in epoch 1 to see the accuracy approaching 0.9999, and the accuracy can be seen between 0.9998 and 1 through epoch 15. Loss values also dropped significantly at epoch1, with a tendency to rise briefly at epoch 7 and then fall again. In conclusion, we can see that the loss value varies between 0 and 0.0007. The Loss value defines the error between the output value and the correct answer, which quantifies the ANN output value to some extent corresponding to the correct answer. The loss function, which is primarily used in Deep learning, uses the sum of error squares (regression) and the cross entropy error (classification). This model uses cross entropy as a classification model to derive loss values.

The verification was conducted through a test set, which is 30% of the total data, and the actual data was classified as good, but 1,013,071 were classified as good, and the actual data was defective, but two were classified as good.

**Figure 6:**
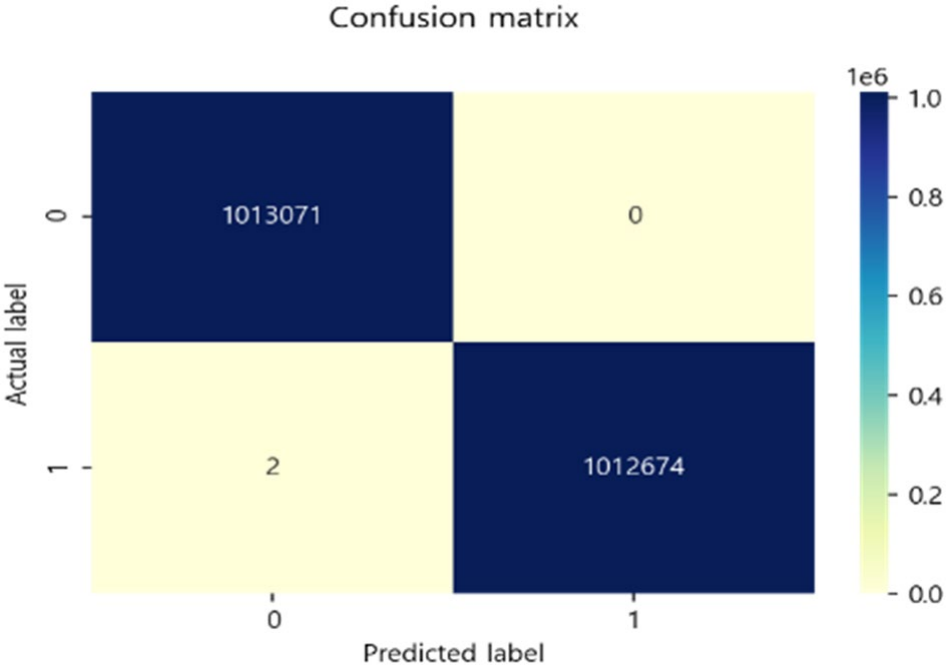
Confusion matrix of ANN.

As in the Confusion matrix in Fig. 6, there were no actual data that were good but judged to be bad, and 1,012,674 were classified as bad when the actual data were bad. It can be seen that it is classified about 100 percent.

The result of applying the fuel tank raw data (44,924 defective products 5,211) to ANN was approximately 47% (see Table 5). The accuracy of the model appeared as a data imbalance, balancing the data, and then applying the model to derive the accuracy again.

**Table 5:** Accuracy of applying fuel tank data to ANN model.

Data	State	Precision	Recall	F1-score	Support
Raw data	Good	1	0.41	0.58	44,924
	Fault	1	1	1	5,211
	Accuracy	–	–	0.471	50,135
Over sampling (SMOTE)	Good	1	0.41	0.58	44,924
	Fault	0.63	1	0.77	44,924
	Accuracy	–	–	0.71	89,848

**Figure 7:**
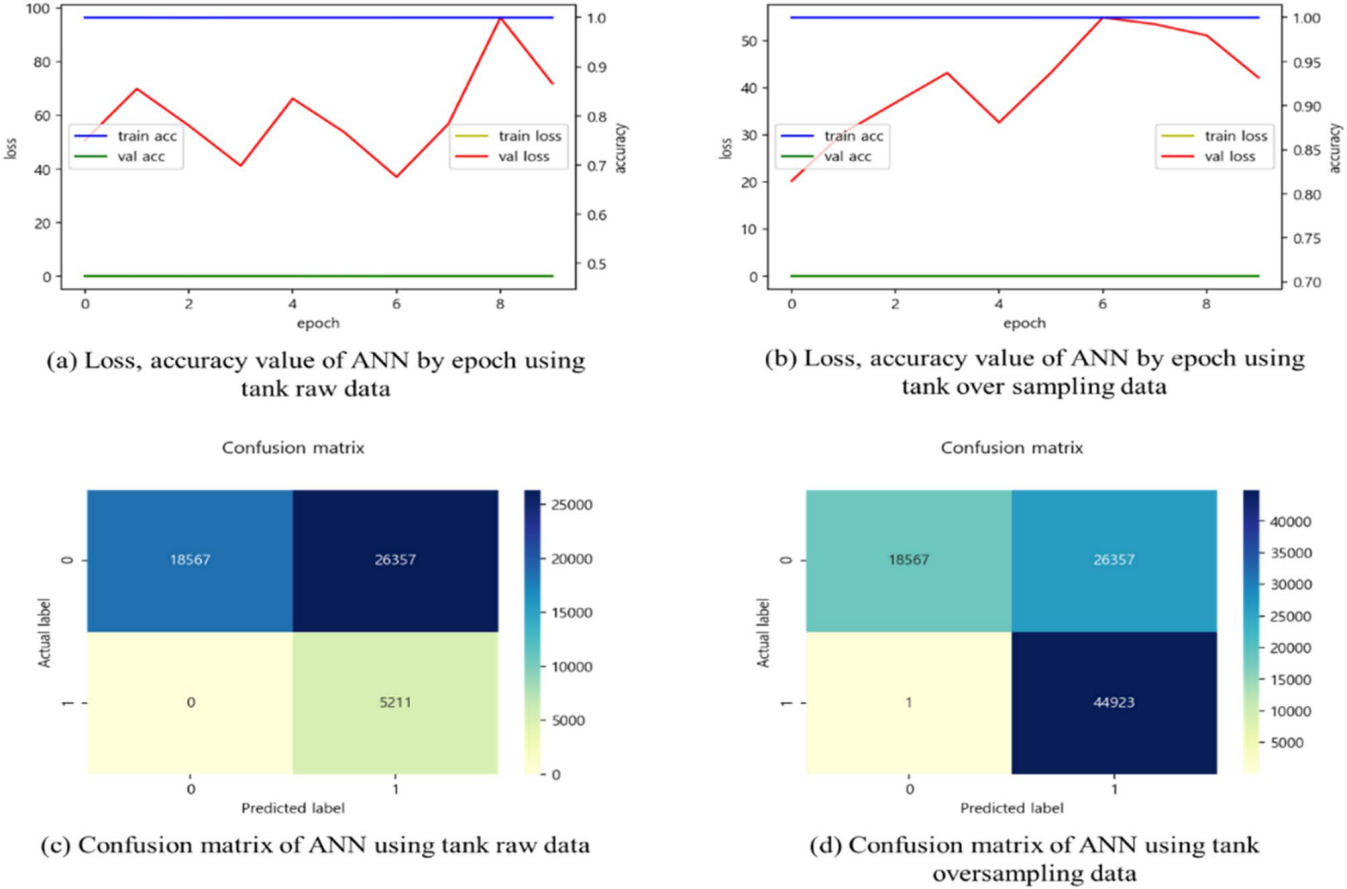
Loss, accuracy, and confusion matrix comparison result between tank oversampling data and tank raw data in ANN model.

Figure 7 is a content that applies fuel tank data to a learning model that utilizes the entire data, and the accuracy continues to converge to 0.47 values. There was an imbalance between good/bad data in the test set, so after performing oversampling with SMOTE, the model was performed again, and the model accuracy was improved by around 24%. In addition, the Loss function also decreased from about 100 to about 50 per 8 epoch. However, the accuracy was lower than that of Logistic regression or Ensemble models. Using fuel tank data (a total of 50,135) as test sets, verification showed that the actual data is good, but the number of good products was 18,567 and the actual data was poor, but there is no good products. The actual data is good, but 26,357 were judged to be defective, and 5211 were classified as defective when the actual data were defective. Classification can be confirmed to be performed incorrectly.

### Comparison result of each model

Using the fuel tank data (50,135) as a test set, the comparison of each model showed that the optimal scaling method for Logistic regression, Ensemble models was a min–max method, which resulted in nearly 100% accuracy when classifying the test set of tests. Standardization has achieved an accuracy of more than 70% except for Random Forest. On the other hand, when classification was performed via the ANN model, approximately 71% accuracy was derived (approximately 47.1% before oversampling), despite oversampling. Therefore, the optimal model for casting parameters showed that the machine learning model performs better than the ANN model. Furthermore, it was derived that the selection of scaling techniques is important because machine learning has a large difference in accuracy according to the data scaling method. The accuracy comparison table for each model is shown in Table 6.

**Table 6:** Accuracy comparison table of each model to which the test set is applied.

Model		Accu-racy	Accuracy applied scaling method			
			Standardization (PCA4)	Normalization (PCA 4)	Robust (PCA 1)	Min–max (PCA 2)
Logistic regression		–	0.99991	0.65445	0.57090	1
Ensemble	Random forest	–	0.55102	0.49893	0.49996	1
	K-nearest neighbors	–	0.80741	0.68534	0.49993	1
	AdaBoost	–	0.82411	0.56419	0.49741	1
	Gradient boosting	–	0.70925	0.44071	0.49804	1
	Voting	–	0.84246	0.48208	0.49804	1
ANN	Raw data	0.471	–	–	–	–
	Over sampling	0.71	–	–	–	–

## Conclusion

In die casting manufacturing sites, the management of casting parameter conditions is paramount for quality improvement. Despite this importance, there is no systematic method because it is managed through the producer’s experience. Therefore, this study established a model to simulate and cope with casting parameter conditions using machine learning/deep learning techniques to solve this part. Two die casting companies that manage similar casting conditions were selected and the product line was classified using approximately 2 million data. We sampled fuel tank data from this family and used it as a test set to validate the model. When establishing a machine learning model utilizing die casting casting parameters values, processing of data scaling and data imbalance has a significant impact on model accuracy. Among the data scaling techniques, results have been obtained that it is appropriate to utilize min–max scaling techniques, and that oversampling SMOTE techniques are valid for insufficient poor data. As a result, it has been demonstrated that using Ensemble machine learning models can be more versatile across product categories compared to using ANN machine learning models. It was derived as the optimal method to utilize the machine learning model logistic regression and ensemble techniques by utilizing min–max scaling and SMOTE techniques. As in previous studies^12,13^, it was found that traditional machine learning algorithm perform better on structured data, and this was also found to be valid for data in the die casting field. Currently, small and medium sized manufacturing companies (die casting) smart factories and AI projects are being led by the government. However, the current site feels the need to introduce AI, but the guidelines on where it can be introduced and what problems can be solved have not been properly established. In addition, quality management of small and medium sized manufacturing companies is the most important management factor, and most of them are currently managed through visual and experience. In addition, requirements for introducing IT systems are often poorly defined. In order to solve this problem, the analysis model establishment and process conducted in this study can be a guideline for small and medium sized manufacturing companies in introducing AI and promoting smart factory enhancement projects. In addition, it is thought that it will be a study that can lay the foundation for problem definition and data collection system to utilize big data and AI analysis in the future.

In performing data analysis, defining problems and collecting data suitable for problem solving exist due to various constraints at the manufacturing site. The die casting casting parameters analysis performed in this study had different protocols for different facilities, which made it difficult to derive models by standardizing various facilities as data are collected in different forms. Although die casting companies perform production with three major facilities (TOYO, Toshiba, UBE), they have different collection systems (protocols) and errors depending on the version of the facility, the equipment maker. It seems that a die casting standard machine learning and deep learning model can be secured only when standardization work is carried out first. In addition, the current poor data is limited to being the optimal model because it preheats the facility before production and contains a number of bad data generated by adjusting casting parameters. This study can be derived that die casting companies should establish machine learning and deep learning models considering these matters when building smart factories in the future.

## Discussion

### Limitation

We considered applying system and machine learning through overall lifecycle management; however, implementing unit product tracking for the entire lifecycle management required changes to the mass production site’s workflow. This presented a practical issue, as it clashed with the existing responsibilities of production site personnel and faced significant resistance to implementation. Consequently, we limited the scope to the die casting process for implementation. As a result, the system established in this study takes a form closer to point of production system (POP) rather than MES. Future refinement and continuous communication with the production site are essential to address this issue. Developing a quality prediction model that encompasses aspects from raw material import to post processing emerged as a challenge to be tackled.

The data acquisition of die casting casting parameters performed in this study has limitations due to the different forms of data acquisition resulting from the different protocols for each equipment, which did not consider various equipment standardization issues. Due to this problem, the study was limited to TOYO equipment from two companies. Even TOYO equipment can have errors depending on the version and age of equipment, which can cause difference in the data. Therefore, it is considered that data collection standardization work should be performed first to secure the standard machine learning and deep learning models for die casting. In the future, research will be conducted on the processing of die casting casting parameter standard data protocols, and once this content is secured, the methodology will be reapplied and verified.

In addition Performing PCA not only makes it difficult to understand the meaning of the resulting variables but also introduces limitations, such as the challenge of using a single data point from the Test set along with the existing Train set, which can lead to issues in accordance with data distribution trends. Nonetheless, despite these drawbacks, PCA was employed for a specific reason. In the case of die casting casting parameters, most features have substantial individual impact, and multicollinearity is expected to be prominent. Therefore, instead of traditional feature selection, it was deemed reasonable to extract new independent variables.

However, if multicollinearity is significant, variables derived from feature selection might adequately represent a sufficient number of variables. In this study, the application of PCA results in a limitation where the influential features are not well understood. Leveraging domain knowledge from the field could lead to the identification of appropriate features, potentially resulting in a more accurate and efficient model development process.

The current objective is to reduce the defect rate of the implemented die casting company, which is around 9%, to as low as 1%. Thus, we have chosen not to avoid a slight level of overfitting, as it might be more beneficial for the model to be slightly overfitted to the current production scenario. Furthermore, considering the context of large scale mass production, where frequent design changes are common, it appears that new data would likely require the development of new models.

### Contribution

This study proposes a quality prediction approach based on casting parameters, which are crucial quality factors in the die casting process. In practical scenarios, there is currently no immediate way to assess the quality of the castings produced in the die casting process. The nature of the product requires chemical bonding time within the product, necessitating quality inspections after post processing (machining). As a result, even defective castings from the die casting process were being processed, incurring significant costs such as tooling and labor.

By applying the die casting process quality prediction model developed in this study, the equipment can directly perform separation and disposal after post processing inspections. This can greatly contribute to reducing post processing costs related to defects. Through the process demonstrated in this study, introducing a quality prediction model into the die casting process is suggested, supporting the significant goals of enhancing quality and reducing costs in the die casting manufacturing field.

## Data Availability

If you need the raw data used in this study, please contact the corresponding author, Sekyoung Youm (sekyoungyoum@gmail.com). Data may be restricted depending on the purpose of use.
